# Circulating MiRNAs as biomarkers of gait speed responses to aerobic exercise training in obese older adults

**DOI:** 10.18632/aging.101199

**Published:** 2017-03-15

**Authors:** Tan Zhang, Tina E. Brinkley, Keqin Liu, Xin Feng, Anthony P. Marsh, Stephen Kritchevsky, Xiaobo Zhou, Barbara J. Nicklas

**Affiliations:** ^1^ Department of Internal Medicine, Section on Gerontology and Geriatric Medicine, Sticht Center for Healthy Aging and Alzheimer's Prevention, Wake Forest School of Medicine, Winston-Salem, NC 27157, USA; ^2^ Center for Bioinformatics and Systems Biology, Department of Radiology, Wake Forest School of Medicine, Winston-Salem, NC 27157, USA; ^3^ Department of Otolaryngology, Wake Forest School of Medicine, Winston-Salem, NC 27157, USA; ^4^ Department of Health and Exercise Science, Wake Forest University, Winston-Salem, NC 27109, USA

**Keywords:** circulating miRNA, aerobic exercise, gait speed, inter-individual variation, older adults

## Abstract

Gait speed is a useful predictor of adverse outcomes, including incident mobility disability and mortality in older adults. While aerobic exercise training (AEX) is generally an effective therapy to improve gait speed, individual responses are highly variable. Circulating microRNAs (miRNAs) may contribute to inter-individual changes in gait speed with AEX. We examined whether plasma miRNAs are associated with gait speed changes (dGaitSp) in 33 obese older adults (age: 69.3±3.6 years, BMI: 34.0±3.1 kg/m^2^, 85% white, 73% women) who performed treadmill walking, 4 days/week for 5 months. Gait speed (baseline: 1.02±0.19 m/s; range of response: −0.2 to 0.35 m/s) was assessed using a 400 meter-fast-paced walk test. Using Nanostring technology, 120 out of 800 miRNAs were found to be abundantly expressed in plasma and 4 of these were significantly changed after AEX: miR-376a-5p increased, while miR-16-5p, miR-27a-3p, and miR-28-3p all decreased. In addition, baseline miR-181a-5p levels (r=-0.40, p=0.02) and percent changes in miR-92a-3p (r=-0.44, p=0.009) associated negatively with dGaitSp. Linear regression combined baseline miR-181a-5p and miR-92a-3p levels showed even stronger associations with dGaitSp (r=-0.48, p=0.005). These results suggest that circulating miR-181a-5p and miR-92a-3p may predict and/or regulate AEX-induced gait speed changes in obese older adults.

## INTRODUCTION

Age-related declines in physical function and subsequent disability are major contributors to morbidity and mortality in older adults [1, 2]. Among those physical function measurements, gait speed has been reported to be a useful predictor of adverse outcomes, including incident mobility disability and mortality in older adults [3-6]. A recent study further supports that walking at a maximum pace might be useful for estimating subjective general health and skeletal muscle mass [7]. Although aerobic exercise training (AEX) is generally effective for improving physical function in older adults, there exists inter-individual variation in responses to standardized AEX interventions [8, 9]. We previously demonstrated an overall benefit of AEX on improvements in fast-paced gait speed over a distance of 400 meters [10, 11]. However, changes in fast-paced gait speed with AEX were highly variable, ranging from a decline of 0.13 m/s to an increase of 0.44 m/s [10]. Results from a previous study further indicate that 0.05 m/s is a small meaningful change in gait speed, while 0.10 m/s is a substantial change in gait speed in older adults, and importantly, small changes in gait speed are detectable in research and clinical settings [12]. Thus, under-standing what physiologic factors contribute to gait speed changes with exercise could be important in tailoring successful interventions to prevent disability. Ideally, identification of novel biomarkers that predict gait speed responses to exercise training will be crucial for clinicians to select intervention regimens in a personalized manner.

Over the last decade, short non-coding microRNAs (miRNAs) have emerged as important regulators in multiple biological processes. These miRNAs are usually about 22 nucleotides long and regulate protein abundance by inhibiting protein translation or enhancing mRNA degradation [13]. miRNAs are involved in epigenetic control of muscle function, including proliferation, differentiation, hypertrophy, and contraction [14-19]. Although some miRNAs are tissue-specific, many can be detected in blood, where they are highly stable and resistant to endogenous RNAses [20-23]. Thus, circulating miRNAs are considered useful disease-specific diagnos-tic biomarkers and promising therapeutic targets [24-27].

Although recent studies demonstrate that circulating miRNAs are associated with aerobic capacity, muscle mass, and muscle strength [28-30], their relationship with physical function and disability is largely unknown. miRNA expression is altered in response to acute, as well as chronic exercise, which may help to elucidate molecular mechanisms that underlie cardiovascular and muscular adaptations [31-33]. Other studies have shown that miRNAs may function as paracrine/endocrine mediators [34], which could be regulated with endurance exercise, indicating a potential role of miRNAs in regulating responses to an exercise intervention [35-37]. However, to our knowledge, the effects of AEX on global miRNA expression profiles in older adults, and the relationship of individual variation in gait speed responses to miRNAs, have not been studied. The present study had two goals: (1) to examine changes in miRNA expression in response to an AEX intervention and (2) to identify circulating miRNA biomarkers related to individual changes in gait speed.

## RESULTS

### High-throughput screening of circulating miRNAs in obese older adults by Nanostring nCounter analysis

**Figure 1:**
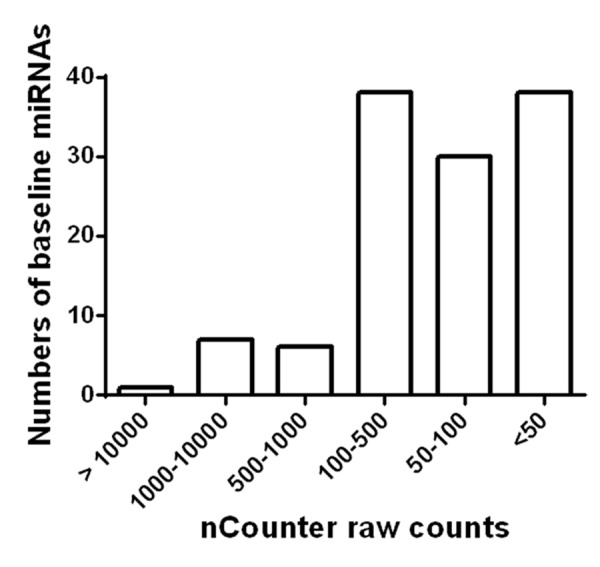
Detection of miRNAs in circulation of obese older adults Based on Nanostring nCounter analysis raw counts, 120 miRNAs were found to be abundantly present in circulation of obese older adults.

Among the 800 miRNAs detected, 120 miRNAs were abundantly expressed in plasma (Figure 1). Among them, only 14 were found to have counts above 500, whereas the majority was between 50 and 500. Details of each miRNA are listed in supplementary Table S1. In addition, circulating levels of 4 miRNAs were significantly changed after AEX (pre vs. post): miR-376a-5p (3.71 ± 2.92 vs. 4.98 ± 2.26, p = 0.004) increased, while miR-16-5p (9.91 ± 0.86 vs. 9.60 ± 0.82, p = 0.039), miR-27a-3p (4.06 ± 2.54 vs. 2.74 ± 2.88, p = 0.022), and miR-28-3p (2.65 ± 2.79 vs. 1.63 ± 2.52, p = 0.043) all decreased.

### Association between gait speed changes and circulating miRNA levels

**Figure 2:**
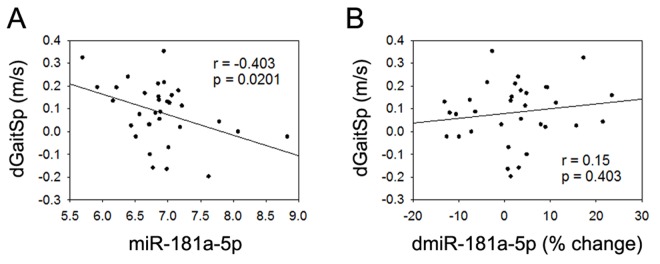
miR-181a-5p and gait speed changes (**A**) Association between changes in gait speed (dGaitSp) and baseline levels of miR-181a-5p; (**B**) Association between changes in gait speed (dGaitSp) and percent changes in miR-181a-5p (dmiR-181a-5p (% change)).

**Figure 3:**
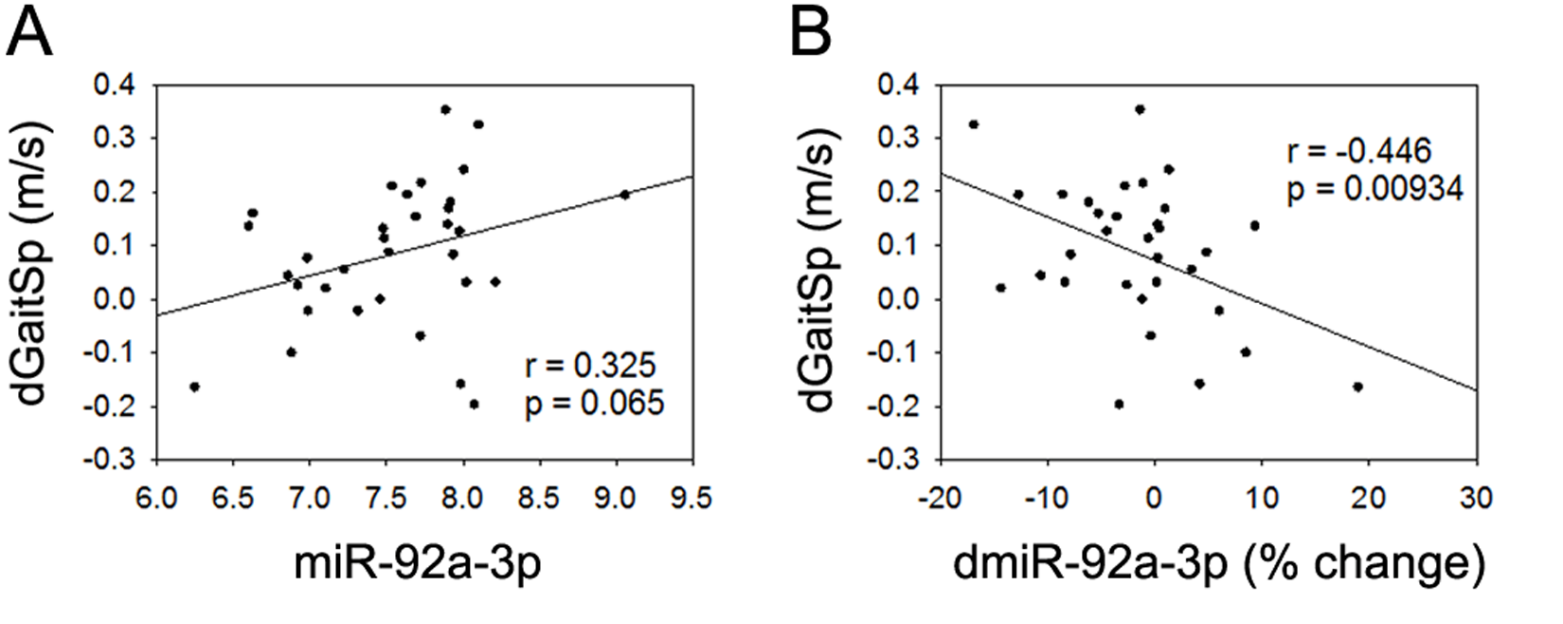
miR-92a-3p and gait speed changes (**A**) Association between changes in gait speed (dGaitSp) and baseline levels of miR-92a-3p; (**B**) Association between changes in gait speed (dGaitSp) and percent changes in miR-92a-3p (dmiR-92a-3p (% change)).

None of the 4 miRNAs that were altered with AEX (miR-376a-5p, miR-16-5p, miR-27a-3p, and miR-28-3p) were associated with gait speed changes in response to AEX (data not shown); thus, we analyzed whether other abundant circulating miRNAs were associated with gait speed changes. We found that changes in gait speed were negatively associated with baseline levels of miR-181a-5p, but not with percent changes in miR-181a-5p with AEX (Figure 2). In addition, changes in gait speed showed a trend for a positive correlation with baseline levels of miR-92a-3p, but negatively correlated with percent changes in miR-92a-3p levels (Figure 3). These associations persisted or became stronger after adjustment for age and gender. Since gait speed changes were not associated with changes in body mass (data not shown), we did not include change in body mass in the model.

### Combined miR-181a-5p and miR-92a-3p baseline levels were strongly associated with gait speed changes with AEX

**Figure 4:**
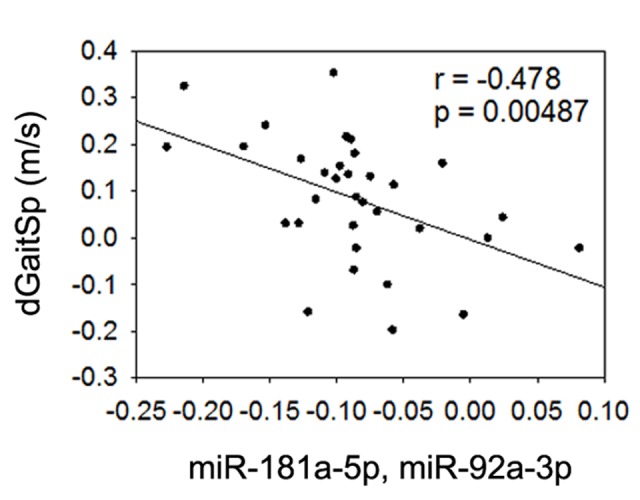
Combined miR-181a-5p and miR-92a-3p and gait speed changes Association between changes in gait speed (dGaitSp) and combined baseline levels of miR-181a-5p and miR-92a-3p.

Given the detected associations or trend of associations between gait speed changes and baseline miR-181a-5p or miR-92a-3p, we next tested if the linear regression combined baseline miR-181a-5p and miR-92a-3p could yield an even stronger association with gait speed changes. Based on a linear combination of the baseline levels of the 2 miRNAs weighted by their regression coefficients as others have used [38], we used a linear combination of miR-181a-5p and miR-92a-3p (−0.208+0.08(miR-181a-5p)-0.057(miR-92a-3p)), and found that the combined baseline level of these miRNAs had an even stronger negative association with gait speed changes than either miRNA alone (Figure 4), even after adjustment for age and gender (p=0.004). This finding implies that combined baseline levels of miR-181a-5p and miR-92a-3p could be useful biomarkers for prediction of gait speed responses to AEX in obese older adults.

### Baseline level adjusted gait speed changes show similar trends of association with miRNAs as those observed between absolute gait speed changes and miRNAs

The above determined associations were based on absolute gait speed changes (dGaitSp (m/s)) after AEX. Given that the initial baseline gait speed varied among individuals (Table 1) and may affect the individual gait speed response to AEX, we further analyzed the association between the identified miRNAs and baseline level-adjusted gait speed changes: dGaitSp (% change) = 100 × (GaitSp^Post-AEX^ – GaitSpP^re-AEX^)/ GaitSp^Pre-AEX^. As shown in Table 2, dGaitSp (% change) shows similar trends of association with miRNAs as that of dGaitSp (m/s). Notably, both dGaitSp (% change) and dGaitSp (m/s) were significantly associated with this linear regression combined variable using baseline levels of miR-181a-5p and miR-92a-3p. This finding confirmed that combined baseline levels of miR-181a-5p and miR-92a-3p are useful for prediction of gait speed responses to AEX in obese older adults, which is independent of the baseline level gait speed, as well as age and gender.

**Table 1:** Participant characteristics at baseline

	Overall (n=33)	Range
**Age (years)**	69.3 ± 3.6	65 – 79
**Gender (M/F)**	24/9	N/A
**Race (White/Non-White)**	28/5	N/A
**BMI (kg/m^2^)**	34.0 ± 3.1	30 – 42
**Self-reported comorbidity (yes/no)**		
Hypertension	19/14	N/A
Diabetes	5/28	N/A
Arthritis	25/8	N/A
Chronic back pain	10/23	N/A
**Medication Use**		
Antihypertensive	23/10	N/A
Lipid-lowering	17/15	N/A
Glucose control	6/27	N/A
Anti-depressant	10/23	N/A
**Baseline gait speed (m/s)** **dGaitSp (m/s)** **dGaitSp (% change)**	1.02 ± 0.19 0.08 ± 0.13 9.26 ± 12.92	0.56 – 1.44 -0.2 – 0.35 -15.24 – 38.89

Table values are mean ± SD or sample size (N). N/A, not applicable.

**Table 2:** Pearson correlation analysis of associations between gait speed changes and plasma miRNAs (n=33)

	miR-181a-5p	dmiR-181a-5p (% change)	miR-92a-3p	dmiR-92a-3p (% change)	miR-181a-5p and miR-92a-3p
dGaitSp (m/s)	R = −0.40 P = 0.02	R = 0.15 P = 0.40	R = 0.33 P = 0.07	R = −0.45 P = 0.009	R = −0.48 P = 0.005
dGaitSp (% change)	R = −0.34 P = 0.05	R = 0.13 P = 0.49	R = 0.31 P = 0.08	R = −0.44 P = 0.02	R = −0.42 P = 0.01

Target genes shared between miR-181a-5p and miR-92a-3p are closely related to biological processes involved in the regulation of neural, skeletal muscle, and vascular function. To better understand the underlying mechanisms related to miR-181a-5p and miR-92a-3p's involvement in gait speed responses to AEX, we next performed analysis of their target genes.

**Figure 5:**
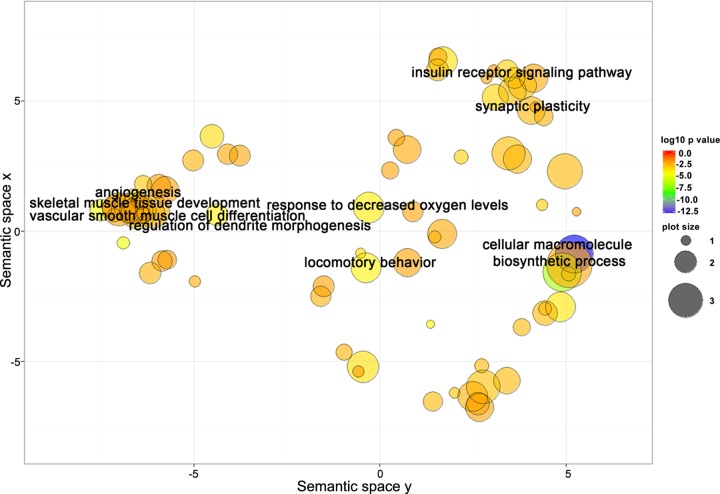
Target genes shared between miR-181a-5p and miR-92a-3p are closely related to biological processes involved in regulation of neural, skeletal muscle, and vascular function Scatterplot showing semantic similarities of enriched GO terms. Bubble color indicates the GO term enrichment p-value. Bubble size indicates the frequency of the GO term in the reference database (EBI GOA database).

Using the multiMiR R analysis, we detected 1,569 target genes for miR-181a-5p and 1,997 genes for miR-92a-3p, 353 of which were common to both. Go analyses yielded 139 BP Go terms for miR-181a-5p target genes, 176 terms for miR-92a-3p target genes, and 104 terms for the common genes. Using REViGO to remove redundant GO terms revealed that a complex set of genes involved in muscle, vascular and neural physiology regulation could be involved in the response to AEX among obese older adults (Figure 5).

## DISCUSSION

The current study provides the first evidence that miR-181a-5p, miR-92a-3p, or their combination, may be useful biomarkers to predict gait speed response to AEX in older adults. Importantly, the identified associations between these two miRNAs and gait speed changes are independent of the baseline gait speed, indicating that these miRNAs may be involved in the regulation of gait speed responses to AEX through mediating their target gene-related signaling pathways, including but not limited to, muscle, vascular, and neural physiology regulation.

These findings add value to the development of ‘personalized medicine’ or ‘precision care’, which is an important concept that has been growing over the recent decades [39]. To date, regular exercise is the most effective therapy for reducing age-related loss of walking speed; however, even though there is non-uniformity of exercise-induced improvement in gait speed among individuals [40], prescription of exercise is often undertaken with a global approach rather than a individualized one [41]. Identification of novel biomarkers that predict gait speed response to therapy is needed before a personalized approach can be used clinically. This will help predetermine if an individual is likely to respond favorably to a certain exercise training mode, or whether a clinical practitioner should prescribe a different therapy.

Given their stability in circulation and the easy application in the clinical practice, circulating miRNAs have been widely studied and used as useful biomarkers for disease prediction, diagnosis, prognosis, and staging [42, 43]. Yet, very few studies have investigated the impact of exercise training interventions on miRNA expression, either in tissue or in the circulation. In addition, research into the role of miRNAs in exercise-induced adaptations has predominantly focused on selected miRNA species with limited numbers based on their reported functions, for instance, inflammation, angiogenesis, hypoxia/ischemia adaptation and metabolism [44-48]. These studies indicate that circulating miRNAs could be affected by different exercise modalities, and thus could be potential useful biomarkers or mediators of exercise mode-specific training adaptations [49, 50]. We simultaneously characterized 800 circulating miRNAs in a sample of obese older adults and our findings provide novel evidence for the potential use of circulating miRNAs for defining gait speed adaptations to AEX in these indi-viduals.

An important difference between this work and previous studies is our use of the Nanostring nCounter analysis platform to measure miRNAs. The FDA-approved Nanostring nCounter analysis platform has been widely used for plasma and body fluid miRNA profiling, which has shown value as a diagnostic marker for various diseases [51-54]. The direct quantitation of miRNAs without any amplification with the Nanostring nCounter platform, and the normalization of the mean abundance of the top 100 expressed plasma miRNAs, make our data more reliable and easier to repeat on a larger scale. Although RT-qPCR based methods are widely used, it is yet unresolved how the data should be normalized [55]. Different studies use different normalization strategies to report miRNA expression levels; consequently, there is no consensus on the optimal approach [56]. However, there is some evidence that the combination of several internal reference miRNA normalizers might be more appropriate than a single universal normalize [57]. We did not perform RT-qPCR for our validation because of the concern that RT-qPCR usually uses an individual miRNA as internal control, and the effects of exercise on this selected internal control is unknown.

Using the Nanostring platform, we identified 120 abundant circulating miRNAs in the plasma. This number is comparable to the number of circulating miRNAs detected from other groups in healthy subjects using different quantitation platforms, including Solexa sequencing, Sanger sequencing, Agilent microarrays and Taqman qPCR array as was reviewed recently [58]. Compared to the reported top 20 plasma miRNAs from 7 healthy individuals measured with Agilent microarrays [58], 9 of our 120 miRNAs overlapped: miR-451a, miR-16-5p, miR-223-3p, miR-4454, miR-21-5p, miR-23a-3p, let-7a-5p, let-7b-5p, and let-7f-5p. This supports the notion that the Nanostring platform used in our study is as efficient as the other miRNA analysis platforms.

We compared our findings with a recent study examining the miRNA plasma signature in response to acute and prolonged AEX training in young individuals [59]. It appears that young and old individuals may use different circulating miRNAs in mediating responses to exercise. For instance, levels of miR-92a were down-regulated after 12 weeks of AEX in the young individuals [59], while we saw no apparent effect of 5 months of AEX on plasma levels of miR-92a in our older subjects. We further compared our findings with another study by Barr et al. [60, 61] that analyzed circulating miRNA responses to a 16-week combined intervention including an energy restriction diet (250 kcal/d) and mixed resistance/aerobic exercise (walking and swimming) (250 kcal/d), focused specifically on 13 preselected plasma miRNAs from middle-aged (35-59 years) individuals. Ten out of the 13 miRNAs they focused on (miR-126-3p, miR-142-3p, miR-223-3p, miR-148b-3p, miR-199a-3p-199b-3p, miR-21-5p, miR-221-3p, miR-423-5p, miR-148a-3p, and miR-140-5p) were also detected by our Nanostring platform analyses in older adults. However, in their study, miR-140-5p, miR-223-3p, and miR-221-3p were modulated by the combined diet and exercise training, whereas we found that miR-376a-3p, miR-16-5p, miR-27a-3p and miR-28-3p were altered by AEX. These differences suggest that individual miRNAs may respond differently based on an individual's age and the type of intervention.

Our study is also the first to associate plasma miRNAs and gait speed changes in response to sustained AEX. Previous studies indicate that miR-181a-5p or its targets (ROPN1L and SLC37A3) are involved in processes highly relevant to exercise response, including immune function, apoptosis, membrane traffic of proteins and transcription regulation [62, 63]. In addition, circulating miR-181a-5p decreases with aging and correlates closely with vascular inflammation and immunity [64, 65]. Similarly, miR-92a-3p plays a key role in regulation of vascular growth [66] and its serum levels increase with aging [67]. Given our limited sample size and the relatively narrow age range of our participants, we did not detect an age-related difference in miR-181a-5p or miR-92a-3p (data not shown). However, our study shows for the first time that signatures of combined baseline levels of miR-181a-5p or miR-92a-3p could be useful noninvasive biomarkers to predict gait speed responses to AEX in obese older adults. It is worth noting that gait speed changes were not associated with any of the four miRNAs that we found to be changed with AEX (miR-376a-3p, miR-27a-3p, miR-16-5p, and miR-28-3p). In fact, plasma miRNAs associated with changes in gait speed do not appear to be affected by AEX, which is consistent with a recent finding that blood-born miRNA patterns that are useful as biomarkers are not necessarily altered by overall fitness and exercise [68]. Therefore, we propose that the miRNA target genes and consequently their related signaling pathways might be regulated differentially, and may subsequently lead to inter-individual variation in reponses to AEX. Further mechanistic studies on changes in miRNA-regulated target gene networks in muscle and other tissues/systems could help detect novel targets as potential useful tools to adjust physiologic adaptations to AEX in older adults.

### Limitations and future directions

Our study has some limitations. Although most of the skeletal muscle-enriched myomiRs [69] (e.g. miR-1, miR-133a, miR-133b, miR-206, and miR-499) were detected by real-time PCR analysis in circulation after various exercise interventions [48, 49, 70-72], none of them were detected in plasma in our assay. This could have been limited by the sensitivity of Nanostring technology, the relatively lower amount of myomiRs released into circulation, or their higher uptake into recipient cells in other tissues. Without skeletal muscle and other tissues collected in parallel with the plasma in our study, we cannot fully interpret regulation of those specific myomiRs. Similarly, the source and target recipient cells of circulating miR-181a-5p and miR-92a-3p could not be further analyzed and verified. Our target gene prediction and interaction analyses indicated that the predicted miR-181a-5p, miR-92a-3p target genes are closely related to regulation of muscle, vascular and neural physiology, and other important pathways relevant to the physiological response to exercise, including but not limited to apoptosis, transcription regulation, and response to oxygen levels [62]. Yet, without examining skeletal muscle samples, we could not determine the direct or indirect physiological effects of those circulating miRNAs on the skeletal muscle systems. Thus, the current experimental design and data do not provide definitive evidence that miR-181 and miR-92a regulate AEX-induced gait speed changes in obese older adults. It also does not provide a thorough understanding of the molecular pathways that are possibly involved in this gait speed response, which will be the direction of our future mechanistic study.

Notably, we observed no significant associations between several other measured variables (chair rise time, short physical performance battery, blood lipid levels, VO2max, glucose levels, or body composition) and gait speed changes. This indicates that although these metabolic and physiological changes are important responses to aerobic training, they are not necessarily closely related with gait speed changes in obese older adults. The identified miRNAs thus are likely involved in other signaling pathways that regulate gait speed response to AEX in obese older adults, such as cytokine/inflammatory responses. Since we did not measure the levels of cytokines and other inflammation markers, we cannot rule out those possible mechanisms at this time. In addition, all participants in this study completed the intervention with high compliance (≥80% attendance). Thus, it is unlikely that differences in the dose of training contributed to variation in gait speed and miRNA responses to AEX. Although 4 miRNAs were found to change with AEX, none of them were found to be associated with gait speed changes. This could have been limited by the relatively small sample size in this study. Given that the 4 AEX changed miRNAs have been previously reported to be regulated by exercise at the tissue level (i.e., skeletal and cardiac muscle) [62, 73-78], it is worth doing further analysis in future studies to explore their related molecular mechanisms.

Given the relatively few participants in this study, we cannot rule out the possibility that some of the observed differences and associations were statistically significant simply due to chance. A future larger and more definitive study with a non-exercise control group is necessary to validate miRNA expression changes with aerobic exercise and determine their role in predicting inter-individual variation in gait speed.

## MATERIALS AND METHODS

### Study design and participants

This study included 33 older adults who completed a 5-month AEX intervention with high compliance (≥80% attendance) [10]. Participants were recruited to be 65-79 years of age, obese (BMI = 30-37 kg/m^2^) with a sedentary lifestyle for the past 6 months, not dependent on a cane or walker, not part of another research study, non-smokers, and free of osteoporosis, abnormal kidney function, insulin-dependent or uncontrolled diabetes, or uncontrolled high blood pressure. Body composition, lower extremity function, and fasting lipid and glucose levels were measured as previously described [10, 79]. Prevalent comorbidities were assessed by self-report and medication use. Clinical characteristics of the study participants are shown in Table 1. The study was approved by the Wake Forest School of Medicine Institutional Review Board and all participants provided written informed consent.

### Exercise intervention

Participants performed supervised treadmill walking 4 days per week for 5 months. Exercise sessions progressed from 15–20 minutes at 50% heart rate reserve (HRR, assessed during a graded exercise treadmill test) during the 1^st^ week to 30 minutes at 65–70% HRR by the end of the 6^th^ week and included a 5-minute warm-up, 5-minute cool-down, and light stretching. Heart rate, treadmill grade/speed, exercise duration, and the amount of energy expended were recorded each session to monitor compliance to the exercise prescription.

### Gait speed

Gait speed was assessed with a fast-paced 400-meter walk test [80]. The test-retest reliability and validity of the 400-meter walk test in middle-aged and older adults have been reported previously [81, 82]. Participants were instructed to walk the 400-meter distance (10 laps on a flat indoor surface 20 m in length) as quickly as possible. Time to complete the walk was recorded in seconds. Standardized encouragement was given every lap.

### RNA extraction from plasma samples

Blood samples were collected into EDTA-treated tubes in the morning after an overnight fast before and 36-48 hours after the last intervention bout of AEX. Plasma was isolated by spinning blood at 2,000 × g for 20 min at 4 °C and stored at −80°C for later analysis. After thawing, the plasma was centrifuged at 2,000 × g for 10 min at 4 °C to remove debris and deplete platelets, and 500 μl supernatant was used for total RNA extraction using kit #5100 (Norgen Biotek, Thorold, Ontario, Canada) according to the manufacturer's protocol. Eluted total RNA (200 μl) was precipitated overnight with sodium acetate/ethanol with a linear acrylamide carrier (Ambion, Austin, TX, USA) for maximum nucleic acid recovery. RNA pellets were concentrated in 15 μl ddH_2_O; 3 μl samples were submitted for profiling on the multiplexed nCounter platform (Nanostring Technologies, Seattle, WA, USA).

### MiRNA expression profiling with Nanostring nCounter analysis

RNA samples were prepared by ligating a specific DNA tag (miR-tag) onto the 3' end of each mature miRNA, according to the manufacturer's instructions. Assay Kit NS_H_MIR_V2.1 (Nanostring Technologies) was used to anneal miRNAs to target specific barcode probes. No amplification was required. Excess tags were removed by restrict digestion at 37 °C. Hybridizations were carried out by combining 5 μl of each miRNA-miR-Tag sample with 20 μl of nCounter Reporter probes in hybridization buffer and 5 μl of nCounter Capture probes (for a total reaction volume of 30 μl) at 65°C for 16–20 hours. Excess probes were removed using a two-step magnetic bead-based purification on the nCounter Prep Station. Abundance of specific target molecules was quantified by imaging the immobilized fluorescent reporters in the sample cartridge with a CCD camera and counting the individual fluorescent barcode probes for each miRNA target (800 human miRNAs) using the nCounter Digital Analyzer. Data were normalized to the top 100 expressed miRNAs [83]. The background was corrected using the nSolver software package. A 30.21 count threshold was set using negative controls based upon the equation: mean + 3× SD. MiRNAs with more than 70% counts below this threshold were excluded.

MiRNA target gene prediction and interaction analysis The multiMiR R package with retrieval of miRNA-target interactions from 14 external databases was used to predict target genes. Furthermore, we performed enrichment analysis for gene ontology using the TopGo package. For miRNA targeted gene sets, Fisher's exact test was implemented, and the enriched Biological Process (BP) Go terms were selected based on a p-value <0.01. The GO list was further summarized using REViGO, which removed redundant GO terms. Data were visualized in semantic similarity-based scatterplots.

### Data analysis

MiRNA data were analyzed using the nSolver software (Nanostring Technologies) and SPSS software from IBM (Armonk, NY, USA). MiRNA raw counts were log2 transformed and all data were expressed as mean ± standard deviation (SD). The Student's t test was used to compare continuous variables. Categorical data were compared using the Chi-square test. Pearson product-moment correlation was used to measure the strength of the association between pairs of variables. A linear combination of identified miRNAs was also calculated to further assess associations with gait speed. Analysis of covariance was used to determine if associations between gait speed and miRNAs were independent of age and gender. A p-value ≤0.05 was considered statistically significant.

## SUPPLEMENTARY MATERIALS AND TABLE


