# Supplementary material for: Circulating MiRNAs as biomarkers of gait speed responses to aerobic exercise training in obese older adults
Source: Aging (Albany NY). 2017 Mar 15;9(3):900–12. doi: 10.18632/aging.101199 (PMC5391238; doi:10.18632/aging.101199)
Supplement: Supplementary file 1 [file aging-09-900-s001.pdf]

Table S1. List of 120 abundant miRNAs in plasma at baseline from 33 older adults

miRNA raw counts	Numbers in group	miRNAs
> 10000	1	miR-451a
1000-10000	7	let-7g-5p, miR-126-3p, miR-142-3p, miR-16-5p, miR-191-5p, miR-223-3p, miR-4454
500-1000	6	let-7a-5p, miR-106a-5p-17-5p, miR-15a-5p, miR-22-3p, miR-520f, miR-720
100-500	38	let-7b-5p, let-7f-5p, let-7i-5p, miR-106b-5p, miR-130a-3p, miR-146a-5p, miR-148b-3p, miR-150-5p, miR-15b-5p, miR-181a-5p, miR-185-5p, miR-197-3p, miR-1976, miR-199a-3p-199b-3p, miR-199a-5p, miR-19b-3p, miR-20a-5p-20b-5p, miR-21-5p, miR-221-3p, miR-23a-3p, miR-25-3p, miR-26a-5p, miR-26b-5p, miR-27b-3p, miR-29b-3p, miR-302d-3p, miR-320e, miR-324-5p, miR-338-3p, miR-342-3p, miR-374a-5p, miR-378e, miR-423-5p, miR-548aa, miR-570-3p, miR-92a-3p, miR-93-5p, miR-548ai
50-100	30	let-7d-5p, miR-107, miR-122-5p, miR-125a-5p, miR-144-3p, miR-145-5p, miR-148a-3p, miR-149-5p, miR151-a-3p, miR-222-3p, miR-24-3p, miR-2682-5p, miR-302b-3p, miR-30a-5p, miR-30b-5p, miR-30d-5p, miR-30e-5p, miR-337-5p, miR-361-5p, miR-376a-3p, miR-382-5p, miR-409-3p, miR-425-5p, miR-4286, miR-494, miR-514b-5p, miR-544a, miR-574-5p, miR-579, miR-612
<50	38	let-7e-5p, miR-10a-5p, miR-1224-5p, miR-1225-5p, miR-1257, miR-125a-3p, miR-132-3p, miR-140-5p, miR-186-5p, miR-18a-5p, miR-2116-5p, miR-23b-3p, miR-27a-3p, miR-28-3p, miR-29c-3p, miR-301a-3p, miR-32-5p, miR-323a-3p, miR-337-3p, miR-340-5p, miR-367-3p, miR-374b-5p, miR-376c, miR-432-5p, miR-450a_5p, miR-484, miR-485-3p, miR-487b, miR-489, miR-503, miR-518b, miR-520d-5p-518a-5p-527, miR-520h, miR-532-5p, miR-548a-5p, miR-548d-3p, miR-590-5p, miR-98
